# Investigation of and Response to 2 Plague Cases, Yosemite National Park, California, USA, 2015

**DOI:** 10.3201/eid2212.160560

**Published:** 2016-12

**Authors:** Mary Danforth, Mark Novak, Jeannine Petersen, Paul Mead, Luke Kingry, Matthew Weinburke, Danielle Buttke, Gregory Hacker, James Tucker, Michael Niemela, Bryan Jackson, Kerry Padgett, Kelly Liebman, Duc Vugia, Vicki Kramer

**Affiliations:** California Department of Public Health, Sacramento, California, USA (M. Danforth, M. Novak, G. Hacker, J. Tucker, M. Niemela, B. Jackson, K. Padgett, K. Liebman, D. Vugia, V. Kramer);; Centers for Disease Control and Prevention, Fort Collins, Colorado, USA (J. Petersen, P. Mead, L. Kingry);; National Park Service, El Portal, California, and Fort Collins, Colorado, USA (M. Weinburke, D. Buttke)

**Keywords:** *Yersinia pestis*, plague, bubonic plague, septicemic plague, vector-borne infections, rodents, fleas, Yosemite, California, United States, bacteria

## Abstract

In August 2015, plague was diagnosed for 2 persons who had visited Yosemite National Park in California, USA. One case was septicemic and the other bubonic. Subsequent environmental investigation identified probable locations of exposure for each patient and evidence of epizootic plague in other areas of the park. Transmission of *Yersinia pestis* was detected by testing rodent serum, fleas, and rodent carcasses. The environmental investigation and whole-genome multilocus sequence typing of *Y. pestis* isolates from the patients and environmental samples indicated that the patients had been exposed in different locations and that at least 2 distinct strains of *Y. pestis* were circulating among vector–host populations in the area. Public education efforts and insecticide applications in select areas to control rodent fleas probably reduced the risk for plague transmission to park visitors and staff.

## Abstract 2

Rapid interagency investigation and public health response probably reduced risk for transmission to other Yosemite visitors and staff.

Plague is a zoonotic disease caused by the gram-negative bacterium *Yersinia pestis*; the organism’s reservoir is rodents and the vectors are fleas (*1**,**2*). Transmission to humans can occur through bites by infected fleas or through handling *Y. pestis*–infected rodents (*1**,**2*). Epidemics of plague still occur on the continents of Africa, Asia, and North and South America (*3*). Plague was introduced to California in 1900 (*1*,*4**–**6*), where over the next 25 years it caused occasional outbreaks in rats commensally residing with humans in urban areas (*2**,**4**,**6*). Shortly after its introduction, *Y. pestis* moved into wild rodent populations, establishing a sylvatic transmission cycle (*7**,**8*). In subsequent decades, plague spread across California and other western states (*9*) periodically affecting humans (*4**–**6**,**10**–**13*).

The human risk of contracting plague is higher during epizootic transmission when *Y. pestis* is amplified among susceptible rodent hosts (*2*), such as the California ground squirrel (*Otospermophilus beecheyi*), the golden-mantled ground squirrel (*Callospermophilus lateralis*), and certain chipmunk species (*Tamias* spp.) (*2**,**3**,**14**,**15*). Higher mortality rates among these animals lead to the release of infectious fleas into the environment (*2*). The California ground squirrel plays a major role in human exposure in California because its predominant flea species, *Oropsylla montana*, is a competent *Y. pestis* vector (*1**,**2*) that is often abundant on this rodent and in its burrows (*16*) and will readily bite humans (*1**,**11*). Since the 1980s, evidence of *Y. pestis* transmission in rodents in the Sierra Nevada mountains has been generally restricted to locations at elevations >1,200 meters (California Department of Public Health, unpub. data, 1983–2015). Despite ongoing sylvatic transmission, human plague remains rare in the western United States (*17**–**19*), including in California, where no cases have been confirmed since 2006 (*20**,**21*).

During the summer of 2015, the Los Angeles County Department of Public Health (LACDPH) and the Georgia Department of Public Health reported 2 cases of plague in persons who had recently travelled to Yosemite National Park (Yosemite). The California Department of Public Health (CDPH), in collaboration with the US Centers for Disease Control and Prevention (CDC) and the National Park Service (NPS), investigated the increased *Y. pestis* transmission in Yosemite. We summarize the epidemiologic, laboratory, and environmental findings and the public health response.

## Methods

### Epidemiologic and Laboratory Investigation

We defined a case of plague as clinically compatible illness and isolation of *Y. pestis* from a person with a history of travel to Yosemite during the 7 days before illness onset. Clinically compatible illness included fever, headache, chills, and malaise in conjunction with regional lymphadenitis, septicemia, or pneumonia (*22*). Patients were identified by their county or state health department and reported to CDPH or CDC.

Diagnosis of plague was made after PCR testing of clinical specimens, including blood and bubo aspirates; Laboratory Response Network assays and culture were used. Recovered isolates were confirmed as *Y. pestis* by bacteriophage lysis (*23*). For whole-genome multilocus sequence typing (MLST), DNA extracted from *Y. pestis* isolates was sequenced by using the PacBio RS II platform and sequence reads were assembled by using a hierarchal genome assembly process (Pacific Biosciences, Menlo Park, CA, USA). Allele calls for 3,979 *Y. pestis* open reading frames (ORFs) (4,046,060 bp) and cluster analyses were performed as described (*24*).

Local and state public health officials interviewed patients with confirmed cases and their family members who had traveled with them. Respondents were asked about their illness history, travel, activities, and interactions with rodents in and around the Yosemite area during the week before illness onset.

### Environmental Investigation

The environmental investigation was prioritized by patient travel itineraries and historical evidence of *Y. pestis* transmission at these locations or in similar habitats. To assess the scope of *Y. pestis* transmission and the potential exposure risk for visitors and park personnel, the investigation was expanded to include additional locations in Yosemite. At prioritized locations, visual risk assessments were conducted to evaluate the presence and abundance of rodents, the type of human activities in the area, and the potential for human exposure to infective fleas (*25*). In areas with suspected *Y. pestis* transmission, a 30 × 30 cm flannel cloth was used to sample fleas from rodent burrow entrances. Rodents were live-trapped for plague serologic testing and flea collection (*25*). For rodent trapping, Sherman (H.B. Sherman Traps, Tallahassee, FL, USA) and Tomahawk (Tomahawk Live Trap, Hazelhurst, WI, USA) live traps were baited once with a mixture of grains and opened either from overnight through the following midday or from early morning through noon. Relative rodent abundance was estimated by calculating the ratio of captured rodents to the total number of traps set and is referred to as the trap success rate. Captured rodents were anesthetized with isoflurane, identified to species, brushed to collect fleas, and subjected to collection of ≈0.1 mL of blood for *Y. pestis* antibody testing. Deer mice (*Peromyscus maniculatus*) collected near structures were euthanized; all other rodents were marked with a numbered ear tag (National Band & Tag Company, Newport, KY, USA) and released near the point of capture. Small mammal handling techniques were reviewed and approved by the CDPH Institutional Animal Care and Use Committee, protocol 2015-14. In addition to live rodent trapping, rodent carcasses reported by Yosemite staff or visitors were collected for testing.

Flea and rodent specimens were tested for *Y. pestis* by CDPH, CDC, and NPS. Blood samples from trapped rodents were sent to CDPH for concurrent testing by passive hemagglutination and passive hemagglutination inhibition to detect antibodies against *Y. pestis* F1 antigen (*23*). All positive passive hemagglutination titers >1:32, the lowest dilution tested, that were negative by passive hemagglutination inhibition were considered positive (*23*). Fleas collected by burrow swabbing, from live-captured rodents, or from rodent carcasses were sent to CDPH or CDC to be identified to species according to standard taxonomic keys (*26*) and to be tested for *Y. pestis*. Fleas of the same species from the same burrow or rodent host were sorted into pools of up to 10 fleas and then homogenized in brain–heart infusion broth by using glass beads and Mixer Mill MM301 (Retsch, Haan, Germany). 

Rodent carcasses were tested at CDPH, CDC, and NPS. Spleen and liver tissues were removed; for direct fluorescent antibody testing, slide touch tissue preparations were incubated with fluorescein isothiocyanate–labeled rabbit anti-F1 antibodies, washed, and then viewed by fluorescence microscopy (*23*). DNA was extracted from flea homogenates and carcass tissues by using the MagNA Pure Compact Nucleic Acid Isolation Kit (Roche Diagnostics, Basel, Switzerland) and amplified by using TaqMan primers and probe targeting the *caf1* gene (*27*). Animal and flea specimens positive by PCR were inoculated onto sheep blood agar plates or onto cefsulodin-irgasan-novobiocin agar plates to enable isolation of *Y. pestis* from contaminated environmental samples (*23*). Isolates were confirmed as *Y. pestis* by bacteriophage lysis and typed by whole-genome MLST (*24*) with the exception that genome sequencing was performed with the MiSeq platform (Nextera XT library preparation, MiSeq Reagent Kit v2, 300 cycle; Illumina, San Diego, CA, USA). Read corrections and assemblies were generated by using SPAdes 3.6 (*28*). All diagnostic tests were performed by using standard negative and positive controls.

## Results

### Laboratory and Epidemiologic Findings

**Figure 1:**
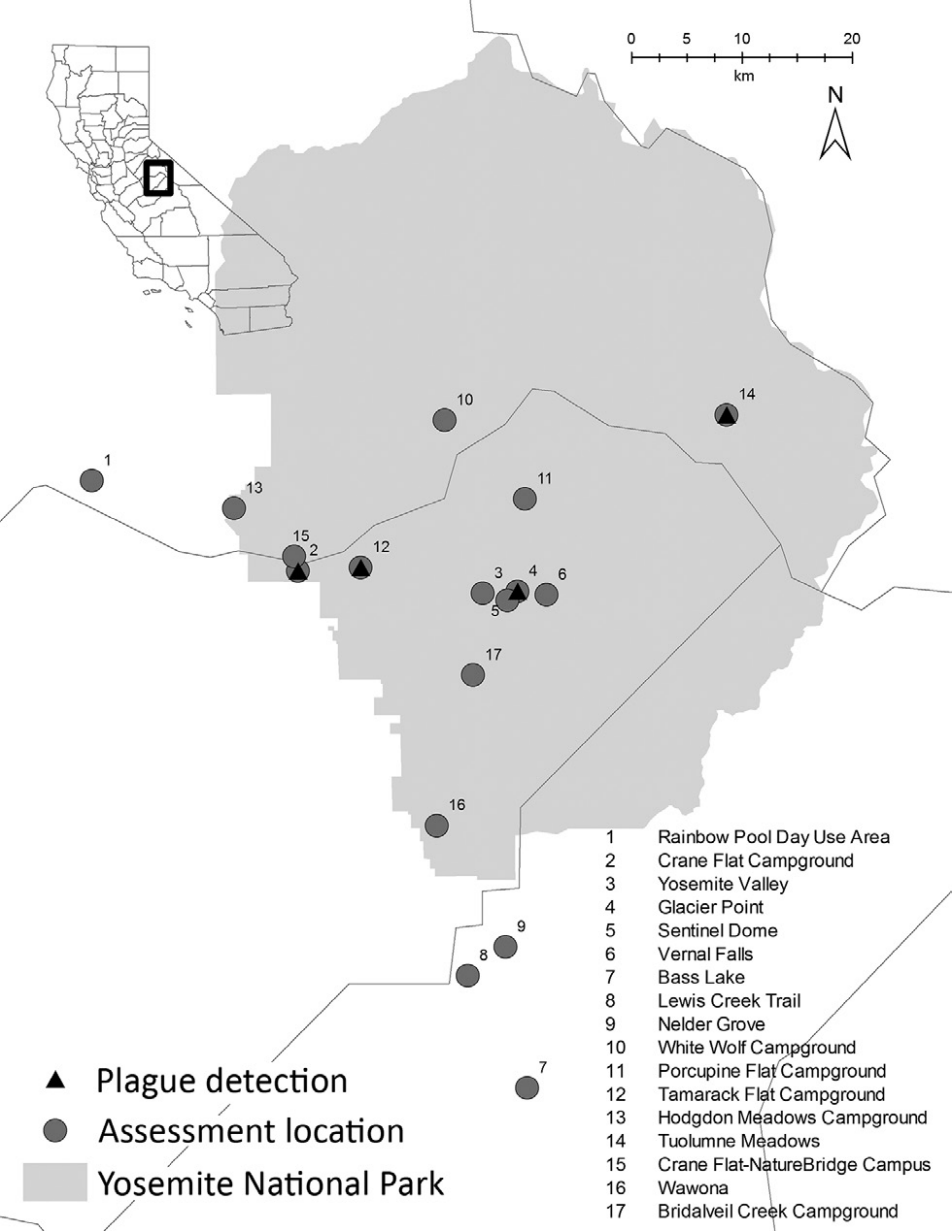
Locations of plague transmission risk assessments in and around Yosemite National Park, California, USA, August–October 2015.

On August 2, 2015, LACDPH presumptively diagnosed septicemic plague for a 14-year-old male resident of Los Angeles County (patient 1) and reported the suspected case to CDPH and CDC (L. Tovar Padua, David Geffen UCLA School of Medicine, pers. comm., 2016 Jan 7). LACDPH later confirmed the diagnosis. The patient became symptomatic on July 18, after camping at Crane Flat Campground in Yosemite July 12–17 and visiting Yosemite Valley and Rainbow Pool Day Use Area (Stanislaus National Forest) during this period (Table 1; Figure 1). He reported that he fed squirrels but did not touch them (L. Tovar Padua, pers. comm., 2016 Jan 7). 

**Table 1:** *Yersinia pestis* transmission risk assessments in and around Yosemite National Park, California, USA, August–October 2015*

Site	Association	Name	Jurisdiction	Elevation, m	Assessment activity*	*Y. pestis* antibody detection
1	Patient 1 visited	Rainbow Pool Day Use Area	Stanislaus National Forest	850	V, B	None
2	Patient 1 visited	Crane Flat CG	Yosemite National Park	1,890	V, B, T, C	Serology +, flea pool +
3	Patients 1 and 2 visited	Yosemite Valley	Yosemite National Park	1,220	H, V, T, C	None
4	Patient 2 visited	Glacier Point	Yosemite National Park	2,190	V, B, T	Serology +
5	Patient 2 visited	Sentinel Dome	Yosemite National Park	2,470	V, B	None
6	Patient 2 visited	Vernal Falls	Yosemite National Park	1,510	V, B	None
7	Patient 2 visited	Bass Lake	Sierra National Forest	1,040	H	None
8	Patient 2 visited	Lewis Creek	Sierra National Forest	1,280	V, B	None
9	Patient 2 visited	Nelder Grove	Sierra National Forest	1,640	V, B	None
10	Expanded investigation	White Wolf CG	Yosemite National Park	2,400	V	None
11	Expanded investigation	Porcupine Flat CG	Yosemite National Park	2,480	V	None
12	Expanded investigation	Tamarack Flat CG	Yosemite National Park	1,940	V, B, T	Serology +
13	Expanded investigation	Hodgdon Meadows CG	Yosemite National Park	1,450	V	None
14	Expanded investigation	Tuolumne Meadows	Yosemite National Park	2,620	V, B, T, C	Serology +, flea pool +, carcass +
15	Expanded investigation	Crane Flat–NatureBridge Campus	Yosemite National Park	1,890	V	None
16	Expanded investigation	Wawona	Yosemite National Park	1,220	C	None
17	Expanded investigation	Bridalveil Creek CG	Yosemite National Park	2,130	V	None

*B, burrow swabbing; C, carcass collection; CG, campground; H, historical review; T, rodent trapping; V, visual assessment; +, positive for *Y. pestis*.

On August 14, the Georgia Department of Public Health presumptively diagnosed bubonic plague for an 18-year-old female resident of Georgia (patient 2) after she had become symptomatic on August 11. On the same day, CDC was notified of the suspected case and later confirmed the diagnosis. During the prior week, this patient had stayed in a rental home in Oakhurst, California, and visited Yosemite Valley, Vernal Falls, Glacier Point, and Sentinel Dome in Yosemite, as well as Nelder Grove, Lewis Creek, and Bass Lake in the adjacent Sierra National Forest (Table 1; Figure 1). Patient 2 reported having observed numerous squirrels in her vicinity at Vernal Falls and Glacier Point but did not report having had any contact with them. 

**Figure 2:**
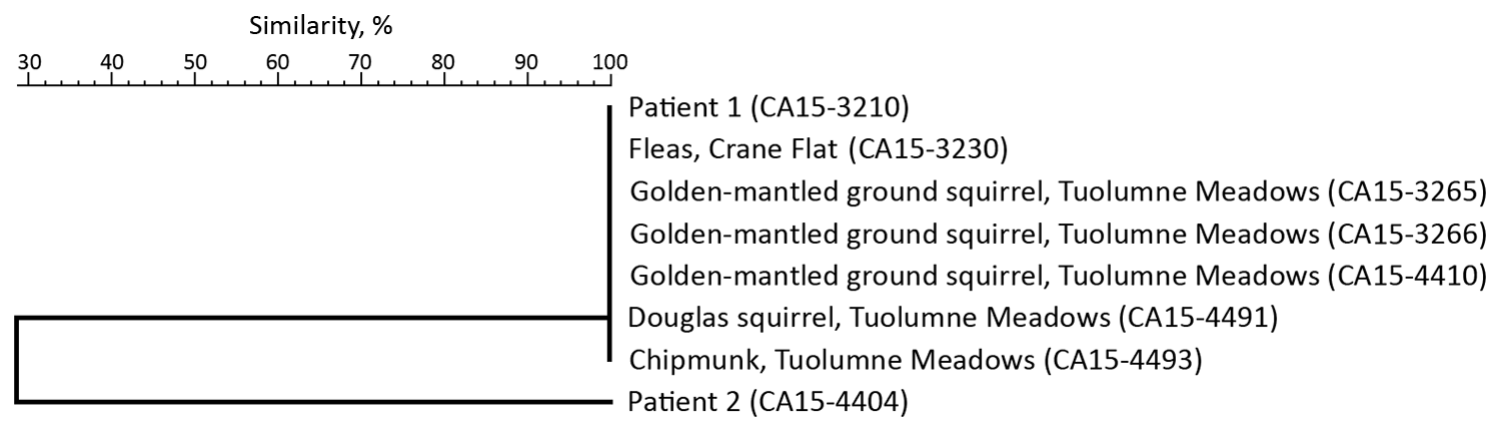
Sequencing results, based on percent similarity, for *Yersinia pestis* isolates from Yosemite National Park, California, USA, August–October 2015.

Neither patient reported seeing dead rodents. Whole-genome MLST showed that the genome sequence of *Y. pestis* isolates from each patient (blood culture from patient 1, bubo aspirate from patient 2) differed at 21 ORFs (Table 2; Figure 2), including 18 single-nucleotide polymorphisms (SNPs). Each patient was accompanied on the trip by family members who did not become ill.

**Table 2:** MLST alleles in whole-genome sequences of *Yersinia pestis* isolates recovered from humans, animals, and fleas, Yosemite National Park, California, USA, August 2015*†

MLST allele, ORF	*Y. pestis* strain CO92 genome position	Mutation type‡	Group 1 isolates§	Group 2 isolates¶
YPCD1.31	22450	SNP	T	C
YPMT1.46	48841	SNP	T	C
YPO0193	211446	6-bp VNTR	–	Loss
YPO0445	467549	1-bp INDEL	–	Deletion
YPO0776	Multiple	9-bp VNTR	Loss	Gain
YPO0894	980089	15-bp VNTR	–	Gain
YPO0968	1072143	SNP	C	T
YPO0976	1084232	SNP	G	T
YPO1332	1498571	SNP	T	C
YPO1422	1617725	18-bp VNTR	Loss	–
YPO1705	1946021	SNP	C	T
YPO2153	2423508	SNP	C	G
YPO2253	2531428	SNP	T	A
YPO2556	2871852	6-bp VNTR	Gain	–
YPO2840	3170905	SNP	T	A
YPO2842	3172167	SNP	T	G
YPO2859	3196474	SNP	A	T
YPO3032	3385894	SNP	A	G
YPO3339	3725154	SNP	T	C
YPO3409	3807578	SNP	T	C
YPO3419	3821161	SNP	C	T
YPO3481	3886839	SNP	C	T
YPO3490	3898668	SNP	T	C
YPO3828	4296699	8-bp INDEL	–	Insertion
YPO4068	4587603	SNP	G	T

*INDEL, insertion/deletion; MLST, multilocus sequence typing; ORF, open reading frame; SNP, single-nucleotide polymorphism; VNTR, variable number of tandem repeats; –, none. †PacBio (Pacific Biosciences, Menlo Park, CA, USA) sequencing of patient isolates yielded an average read length of 15,541 bp with 83,608 average filtered subreads and an average quality and coverage of 84.5% and 202×, respectively. Illumina MiSeq sequencing of flea and animal isolates yielded an average ContigN50 of 40,440 for the 6 assemblies and an average read coverage depth of 84.35×. ‡Gain/loss and insertion/deletion are relative to the CO92 reference genome. All identified SNPs, VNTRs, and INDELs demonstrated at least 10× sequence coverage and 100% of the base calls confirming the mutation. §Group 1; patient 1, flea pool from Crane Flat, animals from Tuolumne Meadows. ¶Group 2; patient 2.

### Environmental Findings

Plague risk assessments were conducted for 9 locations in Yosemite and the surrounding national forests visited by the patients (Table 1; Figure 1). Within the park, 8 more sites were also evaluated for *Y. pestis* transmission and potential risk areas for transmission to humans.

#### Sites Visited by Patient 1

On August 4, the Rainbow Pool Day Use Area (Table 1; Figure 1) was evaluated and deemed to be an area of low risk because of the lack of historical documentation of plague at this habitat and elevation, low abundance of California ground squirrels observed in the day use area, and lack of other diurnal rodent species that are known *Y. pestis* reservoirs in this region. Limited burrow swabbing collected no fleas. A visual evaluation of Yosemite Valley (Table 1; Figure 1) was postponed because it similarly lacked historical documentation of local *Y. pestis* transmission and because no reports of sick or dying rodents from this heavily visited area had been received. The next week, numerous healthy California ground squirrels were noted in Yosemite Valley. At Crane Flat Campground (Table 1; Figure 1), visual assessment and subsequent burrow swabbing suggested recent epizootic activity; California ground squirrel abundance seemed to be very low relative to the number of burrows in the campground, abandoned burrows were noted, and 134 *Y. pestis*–negative fleas were collected from the entrances of 29 (31.5%) of 94 burrows sampled. Carrion flies (*Calliphora latifrons*) were also collected or observed at several burrows. Few chipmunks were observed in the campground, but several Douglas squirrels (*Tamiasciurus douglasii*) were noted. Rodent trapping conducted the following day corroborated the low abundance of rodents (trap success rate 6.9%; Table 3). *Y. pestis* antibodies were detected in 1 (12.5%) of 8 California ground squirrels (Table 4), and a pool of 8 *O. montana* fleas collected from a seronegative California ground squirrel tested positive by PCR for *Y. pestis* (Table 3). Subsequent whole-genome MLST of *Y. pestis* recovered from this flea pool demonstrated 100% sequence identity across all ORFs when compared with the isolate from patient 1 (Figure 2).

**Table 3:** Summary of rodent trapping and seroprevalence of *Yersinia pestis*, by location, Yosemite National Park, August 5–October 22, 2015

Date	Location	Traps set, no.	Rodents caught, no.	Trap success, %	Rodents tested, no.	*Y. pestis*–positive rodents, no.	*Y. pestis* seroprevalence, %
Aug 5	Crane Flat CG	160	11	6.9	11	1	9.1
Aug 25	Glacier Point	100	30	30.0*	27	2	7.4
Aug 25	Crane Flat CG	100	13	13.0*	13	0	0
Aug 25	Tamarack Flat CG	60	11	18.3*	11	1	9.1
Aug 26	Tuolumne Meadows	208	70	33.7*	68	3	4.4
Sep 24	Glacier Point	108	33	30.6	6	1	16.7
Oct 22	Yosemite Valley	175	34	19.4	28	0	0
Total	All	911	202	22.2	164	8	4.9

*Trapping event included overnight hours, which extended the trapping period an additional 12–14 h. CG, campground.

**Table 4:** Summary of *Yersinia pestis*–positive samples, Yosemite National Park, August 5–October 22, 2015*

Location and date	Species	Sample type (titer or test)	Sequence identification no.
Crane Flat CG			
Aug 5	California ground squirrel	Serum (titer 1:64)	NA
Aug 5	California ground squirrel	Flea pool (DFA, PCR, wgMSLT)	CA15-3230
Tuolumne Meadows			
Aug 10	Golden-mantled ground squirrel	Carcass (DFA, PCR, wgMSLT)	CA15-3265
Aug 10	Golden-mantled ground squirrel	Carcass (DFA, PCR, wgMSLT)	CA15-3266
Aug 11	Chipmunk (species unknown)	Carcass (PCR )	NA
Aug 12	Golden-mantled ground squirrel	Carcass (DFA, wgMSLT)	CA15-4410
Aug 14	Chipmunk (species unknown)	Carcass (DFA, culture)	NA
Aug 17	Lodgepole chipmunk	Carcass (DFA, PCR, culture)	NA
Aug 26	Douglas squirrel	Carcass (DFA, PCR, wgMSLT); flea pool (PCR )	CA15-4491
Aug 26	Lodgepole chipmunk	Carcass (DFA, PCR, wgMLST); flea pool (PCR)	CA15-4493
Aug 26	Lodgepole chipmunk	Serum (1:1,024)	NA
Aug 26	Lodgepole chipmunk	Serum (1:512)	NA
Aug 26	Lodgepole chipmunk	Serum (1:128)	NA
Aug 26	Deer mouse	Flea pool (PCR )	NA
Sep 6	Chipmunk (species unknown)	Carcass (PCR )	NA
Sep 8	Chipmunk (species unknown)	Carcass (PCR )	NA
Glacier Point			NA
Aug 25	California ground squirrel	Serum (1:64)	NA
Aug 25	Lodgepole chipmunk	Serum (1:128)	NA
Sep 24	Lodgepole chipmunk	Serum (1:4,096)	NA
Tamarack Flat CG			
Aug 25	California ground squirrel	Serum (1:128)	NA

*Samples analyzed by wgMLST were cultured first. CG, campground; DFA, direct fluorescence antibody; NA, not applicable because sequencing was not performed; wgMLST, whole-genome multilocus sequence typing.

#### Sites Visited by Patient 2

The 7 locations visited by patient 2 were evaluated on August 18 and 19 (Table 1; Figure 1). Bass Lake was not visually assessed because of the historic lack of plague activity in this area. Visual assessments and burrow swabbing at Sentinel Dome, Vernal Falls, Nelder Grove, and Lewis Creek found no obvious indications of *Y. pestis* transmission or increased human risk. Initial evaluation at Glacier Point revealed several abandoned California ground squirrel burrows in close proximity to pathways and picnic areas. From the entrances of the 2 burrows swabbed, 21 fleas were collected. The rodents observed in the area were habituated to humans, and several were noted coming in close proximity to visitors. On the basis of these assessments, Glacier Point was identified as a potential exposure site for patient 2. Rodents were subsequently trapped and tested (Table 3); 1 (7.1%) of 14 California ground squirrels and 2 (22.2%) of 9 lodgepole chipmunks (*Tamias speciosus*) were seropositive (Table 4). All 118 flea pools obtained from Glacier Point, via burrow swabbing or rodent trapping, were negative for *Y. pestis* by PCR.

#### Expanded Investigation

On August 10, NPS was notified that 2 dead rodents were found in the Tuolumne Meadows Campground, ≈25 km from the nearest location visited by the patients (Table 1; Figure 1). During the initial assessment, NPS and CDPH staff observed normal rodent diversity and abundance for this location and no fleas were captured by burrow swabbing. Over the following month, 21 rodent carcasses were collected from the campground and adjacent locations, 17 of which were tested for *Y. pestis*; the remaining 4 were too decomposed for testing. The 2 golden-mantled ground squirrel carcasses collected on August 10 and 8 additional rodent carcasses collected in the campground and surrounding area were positive for *Y. pestis* (Table 4). Flea pools from 2 of the rodent carcasses (1 *Megarthroglossus divisus* flea from a Douglas squirrel, 5 *Ceratophyllus ciliatus mononis* fleas from a lodgepole chipmunk) were also positive by PCR for *Y. pestis* (Table 4). Of the 18 lodgepole chipmunks trapped in this area (Table 3), 3 (16.7%) were positive for *Y. pestis* antibodies (Table 4). A flea pool (3 *Peromyscopsylla hesperomys adelpha* fleas) from a seronegative deer mouse also tested positive for *Y. pestis* (Table 4). Whole-genome MLST of 5 *Y. pestis* isolates recovered from carcasses from Tuolumne Meadows showed that their genome sequences shared 100% sequence identity across all ORFs, compared with the isolates recovered from patient 1 and the flea pool from Crane Flat Campground (Figure 2).

The expanded environmental investigation found evidence of *Y. pestis* transmission at 1 other location (Table 1; Figure 1). Two visual assessments at Tamarack Flat Campground noted a lower than expected abundance and diversity of rodents and numerous abandoned California ground squirrel burrows. Follow-up trapping (Table 3) led to detection of *Y. pestis* antibodies in 1 (20.0%) of 5 California ground squirrels tested (Table 4). Rodent trapping for testing was also conducted in Yosemite Valley in mid-October. None of 13 California ground squirrels and 15 *Peromyscus* mice tested positive for *Y. pestis* antibodies (Table 3). Six rodent carcasses from developed sites in Yosemite Valley and 3 from the Wawona area also tested negative for *Y. pestis*.

### Flea Control

Sites with evidence of recent *Y. pestis* transmission and an increased risk for human exposure were temporarily closed, and rodent burrows were treated with insecticide to reduce flea populations and protect wildlife and human health. The following 5 areas in Yosemite were identified for insecticide treatments: Crane Flat Campground, Glacier Point, Tuolumne Meadows Campground, Tamarack Flat Campground, and the Crane Flat–NatureBridge campus. In total, 16.3 kg of 0.05% deltamethrin was used per label instructions to treat an estimated 3,700 rodent burrows. Although time and logistical constraints precluded pre- and posttreatment flea evaluations at all locations, evidence from limited sampling suggested that the insecticide applications reduced the local flea populations. Before treatment at Crane Flat Campground, 134 fleas had been collected from 94 burrows and the California ground squirrel flea index (total no. fleas on rodents/total no. rodents) was 17.5. After the insecticide application, 58 treated burrows yielded no fleas and the California ground squirrel flea index was 1.0. After insecticide application at Glacier Point, the California ground squirrel flea index declined from 8.1 to 2.7. No pretreatment rodent trapping was conducted at the Tuolumne Meadows Campground to provide comparative flea indices for rodents, but the posttreatment flea index for ground-dwelling rodents was 0.9. Before insecticide application at this site, 80 rodent burrows were marked and sampled, yielding a total of 6 fleas; after treatment, no fleas were found at those same burrows.

### Public Outreach

To further reduce the plague risk for Yosemite visitors and staff, NPS and collaborating agencies initiated an aggressive public education campaign. In 2014, ≈4 million persons visited Yosemite (*29*), and, given that plague cases are rare in the United States, it could not be assumed that most visitors were aware of plague risk or prevention measures. The public education campaign included 3 news releases issued August 6–18, media interviews, and website alerts. The park newsletter, The Yosemite Guide, which was given to persons in every entering vehicle, included information about plague. Placards with plague information were posted at park entrances, locations with confirmed *Y. pestis* transmission, all campgrounds, and many day use locations and trailheads. Educational pamphlets were available to visitors at a variety of locations, including affected campgrounds.

## Discussion

In August 2015, these 2 cases of plague were linked to exposure in the internationally popular Yosemite National Park. The initial public health investigation and response with broad media coverage of the first case led to the rapid recognition and appropriate treatment of the second case-patient (*30*).

The investigation found little overlap in the travel itineraries of the 2 patients, and isolation of distinct strains of *Y. pestis* suggested that at least 2 *Y. pestis* strains were circulating among vector–host populations in the Yosemite area. In the only area visited by both patients, Yosemite Valley, no evidence of *Y. pestis* transmission in rodents was found, and *Y. pestis* has not been detected in the valley’s rodent populations in recent decades (CDPH, unpub. data, 1984–2015). We were able to connect the exposure of patient 1 to epizootic transmission at the campground on the basis of the visual observations at Crane Flat Campground, the positive results for rodent serology and the pool of fleas collected there, and whole-genome MLST analysis of *Y. pestis* isolates from patient 1 and the flea pool. The most likely exposure site for patient 2 was Glacier Point, 20 km away, on the opposite side of Yosemite Valley. Although *Y. pestis*–seropositive rodents were found at this location, we did not detect active infection in rodents or fleas and were therefore unable to directly link the patients’ exposure to this site by whole-genome MLST. Previous findings indicate that *Y. pestis* whole-genome MLST alleles are not rapidly changing and that most detected changes are caused by the more slowly evolving SNPs than by more rapidly changing variable number tandem repeats (*24*). Our results are consistent with those of a previous SNP-based study, which indicated that widespread plague epizootics are caused by multiple *Y. pestis* clones arising independently in small geographic areas (*31*).

The environmental investigation found evidence of *Y. pestis* transmission in disparate locations of the park, including epizootic activity in the Tuolumne Meadows area, ≈41 and 25 km from Crane Flat and Glacier Point, respectively. Evidence of *Y. pestis* transmission in rodents was found at 4 of the 5 areas trapped. Of the 8 species of rodents live trapped in Yosemite, *Y. pestis* antibodies were detected in only 5 (15.2%) of 33 lodgepole chipmunks and 3 (7.3%) of 41 California ground squirrels (Table 5). However, *Y. pestis* was also isolated from golden-mantled ground squirrel and Douglas squirrel carcasses and a deer mouse flea, indicating broader zoonotic involvement.

**Table 5:** Summary of *Yersinia pestis* serology results, by species, Yosemite National Park, August 5–October 22, 2015

Animal (taxonomic name)	Tested, no.	*Y. pestis*–positive rodents, no.	*Y. pestis* seroprevalence, %
Deer mouse (*Peromyscus maniculatus*)	59	0	0
California ground squirrel (*Otospermophilus beecheyi*)	41	3	7.3
Lodgepole chipmunk (*Tamias speciosus*)	33	5	15.2
Golden-mantled ground squirrel (*Callospermophilus lateralis*)	18	0	0
Brush mouse (*Peromyscus boylii*)	6	0	0
Douglas squirrel (*Tamiasciurus douglasii*)	5	0	0
Belding’s ground squirrel (*Urocitellus beldingi*)	1	0	0
Long-tailed vole (*Microtus longicaudus*)	1	0	0
Total	164	8	4.9

During the environmental investigation, serum samples collected from 2 bears killed in Yosemite earlier in the summer were positive for *Y. pestis* antibodies. One bear, killed in July on Tioga Road ≈18 km from Crane Flat Campground, was a cub, indicating that exposure was probably recent. Bears serve as sentinels for plague distribution (*32*), and in recent decades ≈10% of bear blood samples from the Yosemite area have been positive for *Y. pestis* antibodies (CDPH, unpub. data, 1980–2015).

In addition to Yosemite, in 2015, increased *Y. pestis* transmission was evident in other parts of the Sierra Nevada mountains (*33*). Rodent trapping conducted by CDPH in May and June found elevated *Y. pestis* seroprevalence among rodents in Tulare County. A golden-mantled ground squirrel carcass collected in August from Sequoia-Kings Canyon National Park, also in Tulare County, tested positive. In August, evidence of epizootic activity was also detected in Mono and El Dorado Counties.

The plague activity in Yosemite and other parts of California in 2015 was part of a larger regional trend. Although plague is rare in the United States (median 3 human cases/year during 2001–2012) (*17**–**19*), in 2015, the rate increased in western states (16 cases reported to CDC) (*19**,**34*). Synchronous increases in *Y. pestis* transmission in the western United States have been documented previously and are potentially driven by large-scale climatic trends (*35*).

The 2015 findings for Yosemite share some striking similarities with those associated with the only human plague case previously associated with Yosemite (*36*). In 1959, a teenage boy became ill after camping along Yosemite Creek trail, ≈5 km from Crane Flat Campground. Subsequent investigation by CDPH and CDC found evidence of a recent epizootic plague event that had decimated the rodent populations near the campsite. During this investigation, *Y. pestis* transmission was also documented in Tuolumne Meadows and at Lake Tenaya.

The rapid interagency investigation and public health response to these cases probably reduced the risk for plague among Yosemite visitors and staff. Critical risk-reduction measures included expanding the investigation to recreational sites beyond those visited by the patients and localized insecticide treatments at sites with *Y. pestis* transmission. Increased educational efforts informing the public about how to reduce their exposure to the cause of this potentially fatal disease contributed to the early diagnosis for patient 2 and to increased reports of finding dead rodents in the park, which led to detection of *Y. pestis* transmission at additional locations.
